# Evaluation of the anti-cancer efficacy of lipid nanoparticles containing siRNA against HPV16 E6/E7 combined with cisplatin in a xenograft model of cervical cancer

**DOI:** 10.1371/journal.pone.0298815

**Published:** 2024-02-16

**Authors:** Sung Wan Kang, Ok-Ju Kang, Ji-young Lee, Hyejeong Kim, Hunsoon Jung, Hongjoong Kim, Shin-Wha Lee, Yong Man Kim, Eun Kyung Choi

**Affiliations:** 1 Department of Obstetrics & Gynecology, Asan Medical Center, University of Ulsan College of Medicine, Seoul, Republic of Korea; 2 Asan Preclinical Evaluation Center for Cancer TherapeutiX, Asan Medical Center, Seoul, Republic of Korea; 3 EnhancedBio Inc., Seoul, Republic of Korea; 4 Department of Radiation Oncology, ASAN Medical Center, University of Ulsan College of Medicine, Seoul, Republic of Korea; Chapman University, UNITED STATES

## Abstract

**Objective:**

To investigate the anti-cancer efficacy of ENB101-LNP, an ionizable lipid nanoparticles (LNPs) encapsulating siRNA against E6/E7 of HPV 16, in combination therapy with cisplatin in cervical cancer *in vitro* and *in vivo*.

**Methods:**

CaSki cells were treated with ENB101-LNP, cisplatin, or combination. Cell viability assessed the cytotoxicity of the treatment. HPV16 E6/E7 gene knockdown was verified with RT-PCR both *in vitro* and *in vivo*. HLA class I and PD-L1 were checked by flow cytometry. A xenograft model was made using CaSki cells in BALB/c nude mice. To evaluate anticancer efficacy, mice were grouped. ENB101-LNP was given three times weekly for 3 weeks intravenously, and cisplatin was given once weekly intraperitoneally. Tumor growth was monitored. On day 25, mice were euthanized; tumors were collected, weighed, and imaged. Tumor samples were analyzed through histopathology, immunostaining, and western blot.

**Results:**

ENB101-LNP and cisplatin synergistically inhibit CaSki cell growth. The combination reduces HPV 16 E6/E7 mRNA and boosts p21 mRNA, p53, p21, and HLA class I proteins. In mice, the treatment significantly blocked tumor growth and promoted apoptosis. Tumor inhibition rates were 29.7% (1 mpk ENB101-LNP), 29.6% (3 mpk), 34.0% (cisplatin), 47.0% (1 mpk ENB101-LNP-cisplatin), and 68.8% (3 mpk ENB101-LNP-cisplatin). RT-PCR confirmed up to 80% knockdown of HPV16 E6/E7 in the ENB101-LNP groups. Immunohistochemistry revealed increased p53, p21, and HLA-A expression with ENB101-LNP treatments, alone or combined.

**Conclusion:**

The combination of ENB101-LNP, which inhibits E6/E7 of HPV 16, with cisplatin, demonstrated significant anticancer activity in the xenograft mouse model of cervical cancer.

## Introduction

Cervical cancer is a significant global health issue, especially in developing countries where it is one of the primary causes of cancer-related deaths among women [1]. Treatment options depend on the stage and extent of progression and may involve surgery, radiation, chemotherapy, or a combination of these approaches. While surgery is commonly used for early-stage cervical cancer, radiation therapy is preferred for locally advanced diseases [2, 3]. Chemotherapy is often combined with radiation therapy to enhance its effectiveness [4]. However, conventional chemotherapy agents can harm both cancer cells and normal rapidly dividing cells, resulting in severe side effects such as anemia and alopecia [5, 6]. Notable progress has been achieved in cervical cancer treatment through the development of vaccines, adoptive T cell therapy, and immune checkpoint inhibitors such as pembrolizumab and nivolumab, which have demonstrated effectiveness [7–11]. Despite these advancements and ongoing research efforts, overall patient survival rates have not significantly improved, highlighting the need for better treatment strategies [12].

RNA interference (RNAi) has emerged as a potential therapeutic strategy for targeting oncogenes in cancer therapy [13, 14]. In the treatment of cervical cancer, researchers have investigated the use of RNAi to target the E6 and E7 oncoproteins of human papillomavirus (HPV), as these proteins play a critical role in the development and progression of cancer [15, 16]. Targeting HPV E6/E7 with RNAi-based approaches can lead to the accumulation of tumor protein p53 (TP53) and hypo-phosphorylated retinoblastoma (RB), inducing senescence or apoptosis in malignant cells [13, 16–18]. Previous studies have demonstrated improved therapeutic outcomes by combining small interfering RNA (siRNA) targeting E6/E7 with cisplatin or radiation therapy in cervical cancer models [14, 19]. However, the effective delivery of siRNA to the tumor site remains a challenge due to its degradation and limited cellular uptake.

To address this challenge, lipid nanoparticles (LNPs) have been considered a potentially favorable approach for delivering siRNA as they offer protection against degradation and improved cellular uptake [20–23]. LNPs have been successfully utilized in FDA-approved drugs and COVID-19 vaccines [24–27]. However, their potential for treating cervical cancer has not been explored. ENB101-LNPs are engineered ionizable lipid nanoparticles designed for the targeted delivery of siRNA against HPV16 E6/E7 in cervical cancer cells. While this technology has shown efficacy in delivering siRNA to liver cells [28], its effectiveness in cervical cancer treatment has yet to be investigated.

This study aims to evaluate the therapeutic efficacy of ENB101-LNPs, either alone or in combination with cisplatin or radiation therapy, in a xenograft model of cervical cancer.

## Materials and methods

### Cell culture

The CaSki (RRID:CVCL_1100) cell line was obtained from the Korean Cell Line Bank (KCLB) in Seoul, Korea. The cells were cultured in RPMI-1640 medium, supplemented with 10% fetal bovine serum and 1% antibiotic-antimycotic solution at 37°C. The cell line was authenticated using short-tandem repeat (STR) profiling within the last 3 years (S2 File). Mycoplasma contamination was tested using the MycoAlert™ PLUS detection kit (Lonza, Basel, Switzerland). All experiments were performed with mycoplasma-free cells.

### Ionizable LNP preparation

Ionizable LNPs were kindly provided by EnhancedBio Inc. (Seoul, South Korea). LNPs were prepared using the NanoAssemblr Benchtop Instrument (Precision Nanosystems Inc., Vancouver, Canada) according to a previously described method [28]. The lipid components (ionizable lipid, distearoylphosphatidylcholine, cholesterol, and polyethylene glycol lipid at a molar ratio of 42.5:13:43.5:1.0) were dissolved in ethanol, and HPV16 E6/E7 siRNAs (target sequence: 5’-GAC CGG UCG AUG UAU GUC UUG-3’) were dissolved in 50 mM sodium acetate. The final weight ratio of ionizable lipid to RNA was 7.5:1, and the final volume ratio was 1:3. LNPs were formulated by microfluidic mixing of the prepared solutions at a flow rate of 12 ml/min. The resulting LNPs were dialyzed against 1X phosphate-buffered saline (PBS) using dialysis cassettes with a 10,000 molecular weight cutoff (Life Technologies, CA, USA) for 16 hours to exchange the buffer. To characterize the prepared LNPs, dynamic light scattering was used to confirm their size, polydispersity index (PDI), and zeta potential. The encapsulation efficiency of RNAs was measured using the Quant-iTTM Ribogreen Assay (Life Technologies, CA, USA).

### Real-time PCR

To assess the mRNA expression levels of HPV16 E7 and p21, RNA was extracted from cells and tissue using the RNeasy mini kit (Qiagen, CA, USA). Total RNA (1.0 mg) was then reverse transcribed into cDNA following the manufacturer’s instructions (Roche Diagnostics, Basel, Switzerland). The 2^−ΔΔCT^ method was used to calculate the relative expression. E7 and p21 expressions were normalized to that of internal control hypoxanthine phosphoribosyltransferase 1 (HPRT1). qRT-PCR was performed using the LightCycler Real-time PCR Detection system (Roche Diagnostics, Basel, Switzerland). The PCR conditions were as follows: 95°C for 10 min, 45 cycles at 95°C for 10 s, 60°C for 30 s, 70°C for 1 s, and finally 40°C for 30 s. The 2^−ΔΔCT^ method was used to calculate the relative expression. Primers and probes were designed using the online Universal ProbeLibrary Assay Design Center (Roche Diagnostics, Basel, Switzerland). mRNA expressions of HPV16 E7, p21, and HPRT1 were measured using the following primers and probes: HPV16 E7 forward primer: 5’-CCA CTG ATG TCT ACT GTT ATG AGC AA-3’; reverse primer: 5’-CCA GCT GGA CCA TCT ATT TCA-3’ and UPL probe: #63; p21 forward primer: 5’-CGA AGT CAG TTC CTT GTG GAG-3’; reverse primer: 5’-CAT GGG TTC TGA CGG ACA T--3’ and UPL probe, #82; HPRT1 forward primer:, 5’-TGA CCT TGA TTT ATT TTG CAT ACC-3’; reverse primer: 5’-CGA GCA AGA CGT TCA GTC CT-3’; UPL probe #73).

### Western blot analysis

CaSki cells were lysed in RIPA buffer (Thermo Fisher Scientific, MA, USA) with a protease inhibitor (Roche, Basel, Switzerland). Protein concentrations were determined using the PierceTM BCA Protein Assay Kit (Thermo Fisher Scientific, MA, USA), and absorbance measurements were taken at 562 nm on a microplate reader (Tecan, Maännedorf, Switzerland). Protein lysates were separated using SDS-PAGE (8%–15%), and subsequently transferred to polyvinylidene difluoride (PVDF) membranes (Merck, MA, USA).

After blocking the membranes in 5% skim milk in Tris-buffered saline and Tween 20 (TBST) for 1 h at room temperature, they were then incubated overnight at 4°C with the respective primary antibodies against HPV16 E7 (sc-6981, Santa Cruz, TX, USA), p53 (sc-47698, Santa Cruz, TX, USA), p21 (sc-817, Santa Cruz, TX, USA), PD-L1 (13684, Cell Signaling Technology, MA, USA), or β-actin (sc-47778, Santa Cruz, TX, USA), diluted in the blocking buffer. The next day, the membranes were incubated with horseradish peroxidase-conjugated goat anti-rabbit/mouse secondary antibodies (Santa Cruz, TX, USA) as appropriate. After 2 hours at room temperature, signals were developed with an enhanced chemiluminescence solution (Advansta, CA, USA). The band intensities were quantified and analyzed with ImageQuant LAS 4000 imager (GE Healthcare, Buckinghamshire, UK). Relative band intensity was normalized to β-actin and quantified by ImageJ 1.54 open-source software (https://imagej.nih.gov/ij/).

### Flow cytometry

CaSki cells were harvested and blocked with Fc-block antibody (564220, BD Biosciences, CA, USA) and stained with fluorochrome-conjugated antibodies against HLA class I (311404, BioLegend, CA, USA) and PD-L1 (329706, BioLegend, CA, USA) for 30 min. FITC Mouse IgG2a and PE Mouse IgG2b isotype controls from BioLegend (CA, USA) were used in parallel. Dead cells were excluded using a Dead Cell Stain kit (Invitrogen, CA, USA). To assess apoptosis, CaSki cells were subjected to an apoptosis assay using the FITC Annexin V Apoptosis Detection Kit (BD Biosciences, CA, USA). The cells were resuspended and then incubated with FITC Annexin V and Propidium Iodide (PI) for 15 min. Single cells were determined using the FACS Canto II instrument (BD Biosciences, CA, USA), and the data were analyzed using FlowJo software (TreeStar, OR, USA).

### Mice

Female BALB/c slc-nu/nu mice (6 weeks old) were obtained from Japan SLC, Inc. (Shizuoka, Japan). The Institutional Animal Care and Use Committee (IACUC) of the Asan Institute for Life Sciences at the Asan Medical Center approved all animal experiments (for the combination experiment with cisplatin, approval ID: 2021-12-131, approval date 11 May 2021, for the combination experiment with radiation, approval ID: 2022-12-136, approval date 9 May 2022), and the studies were conducted in accordance with authorized guidelines and regulations.

### *In vivo* experiments

The subcutaneous xenograft model was utilized by mixing 5×10^6^ CaSki cells with 50% Matrigel and injecting them into the right hind limb of each mouse. Upon the tumor reaching a volume of 120 mm^3^, the mice were randomly allocated into groups consisting of a blank control, 1 mg/kg ENB101-LNP, 3 mg/kg ENB101-LNP, cisplatin, ENB101-LNP-cisplatin, and ENB101-LNP-cisplatin, with six mice per group. The ENB101-LNP was administered intravenously three times a week for three weeks after mixing with ApoE protein, while cisplatin was administered via intraperitoneal injection once a week for the same duration. To evaluate the efficacy of radiation treatment, we treated the tumors on the right hind limb of the mouse with a single dose of 5 Gy irradiation using a 6-MV photon beam linear accelerator (CL/1800, Varian Medical Systems, CA, USA). Tumor growth curves were used to calculate the tumor inhibition rate, and the mice were euthanized one week after the final treatment for analysis of tumor tissues.

### Histological analysis and immunohistochemistry

Following the last measurement of tumor volume, all transplanted mice were euthanized according to the experimental protocol. The subcutaneous tumor tissues were collected, fixed, and embedded in paraffin for histopathological analysis. Hematoxylin and eosin (H&E) staining was performed, and microscopic images were captured using a microscope (Olympus, Tokyo, Japan). To perform immunohistochemical staining, Tris-EDTA buffer (Vector Laboratories, CA, USA) was used to heat the paraffin-embedded tissue sections for antigen retrieval. Endogenous peroxidase activity was blocked using BLOXALL Endogenous Blocking Solution (Vector Laboratories, CA, USA). The slides were then blocked using a 2.5% normal goat serum blocking solution (Vector Laboratories, CA, USA) for 30 minutes at room temperature before being stained overnight at 4°C with primary antibodies against anti-p53 (ab131442, Abcam, CA, USA), anti-p21 (sc-6246, Santa Cruz, TX, USA), anti-RB (554136, BD Biosciences, CA, USA), and anti-HLA-A (ab52922, Abcam, CA, USA). The sections were incubated with the secondary antibodies against biotinylated mouse or rabbit IgG (Abcam, CA, USA) for 1 hour and with ABC reagents (Vector Laboratories, CA, USA) for 30 minutes at room temperature. Finally, the sections were stained with DAB for 10 minutes, counterstained with 1% methyl green, and then embedded using a mounting medium. The images were scanned using the VS200 digital slide scanner (Olympus, Tokyo, Japan).

### TUNEL staining

To detect apoptotic cells in tissues, we used the TUNEL assay kit from Merck (MA, USA) following the manufacturer’s instructions. Briefly, after deparaffinization and rehydration, sections were incubated with proteinase K (20 mg/mL, Qiagen, CA, USA) for 15 minutes at room temperature. Slides were then rinsed with PBS and incubated with 3% H_2_O_2_ in PBS for 5 minutes at room temperature to block endogenous peroxidase activity, followed by washing with PBS. The sections were then incubated with a mixture of terminal deoxynucleotidyl transferase solution and digoxigenin-dUTP solution in a humidified chamber at 37°C for 1 hour, followed by washing with PBS and incubation with anti-digoxigenin-peroxidase conjugates in a humidified chamber for 30 minutes at room temperature. After an additional wash with PBS, the slides were incubated in DAB and counterstained with 1% methyl green. The number of TUNEL-positive tumor cell nuclei was then counted.

### Statistical analysis

GraphPad Prism software (GraphPad, La Jolla, CA, USA) was used for statistical analysis. Student *t*-test or analysis of variance (ANOVA) was used for intergroup comparison.

## Results

### ENB101-LNP and cisplatin synergistically inhibit cell proliferation through the activation of the p53/p21 pathway and the regulation of human leukocyte antigen (HLA) class I and programmed death-ligand 1 (PD-L1) expression in CaSki cells

**Fig 1:**
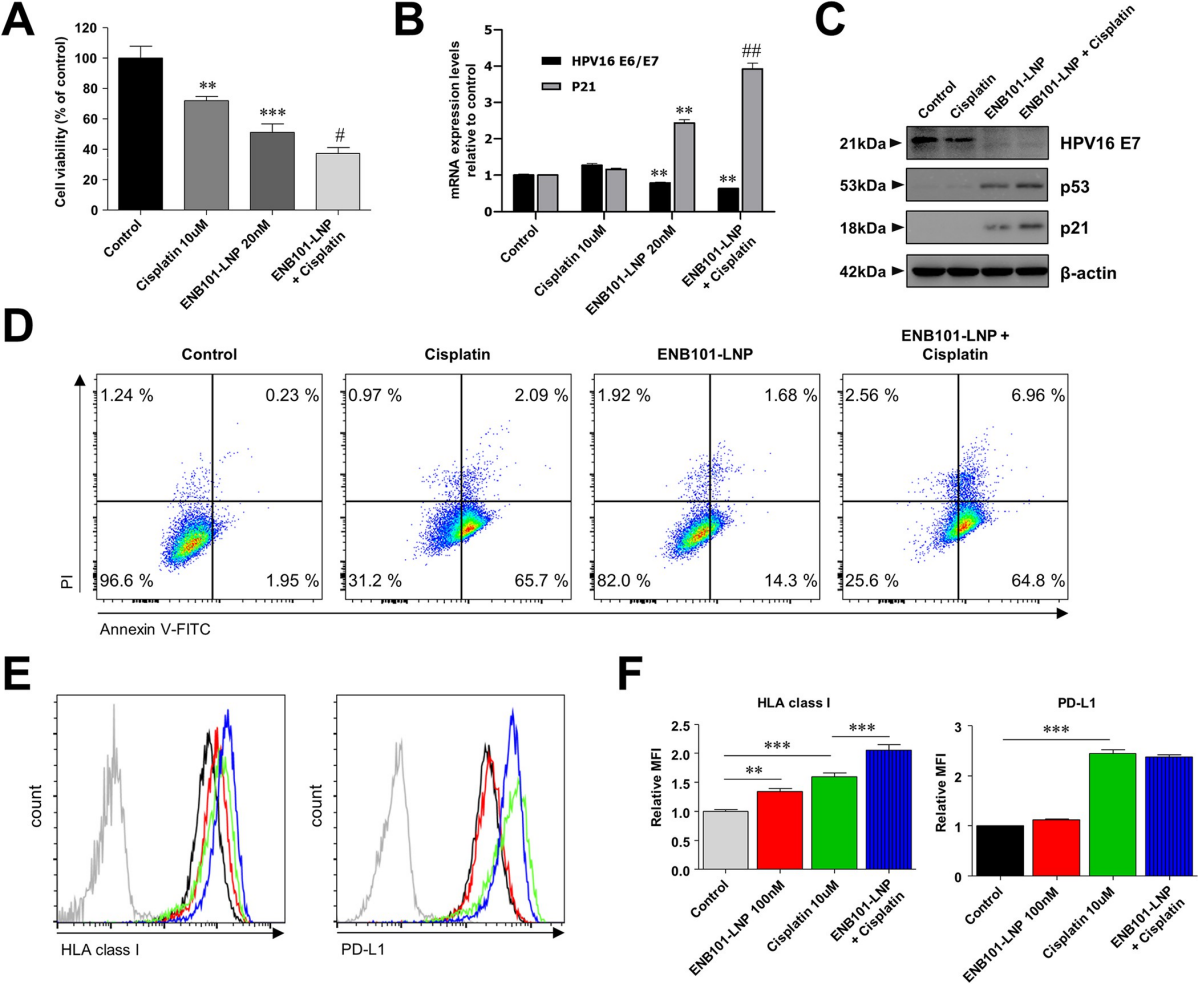
ENB101-LNP and cisplatin synergistically inhibit cell proliferation through the activation of the p53/p21 pathway and the regulation of HLA class I and PD-L1 expression in CaSki cells. CaSki cells were plated in replicates and treated with a PBS control, cisplatin (10 μM), ENB101-LNP (20 nM), or a combination for 24 or 48h. (A) Cell viability was determined using the MTS assay and expressed as a percentage of the control. (B) qRT-PCR analysis of the mRNA levels of HPV16 E7 and p21. Data are mean ± standard deviation; comparisons were made by unpaired *t*-test (** P < 0.01, *** P < 0.001; compared with control, # P < 0.05, ## P < 0.01; compared with ENB101-LNP). (C) Western blot analysis of HPV16 E7, p53, and p21 protein levels. (D) Apoptotic cells were analyzed using flow cytometry with Annexin V-FITC and PI staining. In this analysis: (i) the lower left quadrant corresponds to viable cells, (ii) the lower right quadrant indicates cells in early apoptosis, (iii) the upper right quadrant represents cells in late apoptosis, and (iv) the upper left quadrant denotes dead cells. (E-F) The synergistic effect of ENB101-LNP (100 nM) and cisplatin (10 μM) treatment for 48 h on the expression of HLA class I and PD-L1 in CaSki cells was measured using flow cytometry. (E) Representative histograms of surface HLA class I and PD-L1 expression. (F) The expression levels are quantified as a percentage reflecting the median fluorescence intensity (MFI). MFI data are represented as the mean ± standard deviation of three independent experiments. P values were calculated using one-way ANOVA with Bonferroni’s post hoc test (** P < 0.01, *** P < 0.001).

To evaluate the effectiveness of cisplatin and ENB101-LNP, we treated cisplatin (10 μM), ENB101-LNP (20 nM), or their combination into HPV16-positive CaSki cells. (Fig 1) The cells treated with both cisplatin and ENB101-LNP exhibited the highest rate of cell death (Fig 1A). In the cells treated with cisplatin and ENB101-LNP, the expression rate of HPV 16 E6/E7 mRNA was lower compared to the control, and the expression level of p21 mRNA increased by about 4-fold (Fig 1B). The changes in the levels of HPV E7, p53, and p21 proteins were assayed by immunoblotting. Transfection with ENB101-LNP significantly reduced HPV16 E7 expression and increased the levels of p53 and p21, respectively (Fig 1C and S1 Fig). The ENB101-LNP treatment resulted in a 0.4-fold decrease in the expression level of HPV 16 E7 protein in CaSki cells compared to the control. Furthermore, the combination treatment of ENB101-LNP and cisplatin showed an even greater reduction of 0.2 times compared to the control (Fig 1C and S1A Fig). The cells transfected with ENB101-LNP exhibited an increased p53 and p21 expression compared to the control cells. Additionally, when treated with both ENB101-LNP and cisplatin, these cells displayed higher levels of p53 and p21 proteins than cells transfected solely with ENB101-LNP (Fig 1C and S1B Fig). To assess the induction of apoptosis in CaSki cells by ENB101-LNP and cisplatin, the apoptotic rate in these cells following treatment with both agents was measured using flow cytometry, employing Annexin V/PI staining. Treatment with ENB101-LNP (2.55% ± 1.23), cisplatin (2.24% ± 0.21), or a combination of both (9.03% ± 2.93) resulted in a heightened apoptotic ratio in CaSki cells when compared to control (0.82% ± 0.83). Remarkably, cells treated with the combination of ENB101-LNP and cisplatin showed a significantly higher apoptotic rate than those treated with either agent alone (Fig 1D). These findings indicate that ENB101-LNP, alone or combined with cisplatin, significantly inhibits growth, induces apoptosis, and affects the expression of p53 and p21 proteins.

To evaluate the effect of ENB101-LNP and cisplatin treatments on the expression levels of HLA class I and PD-L1, we conducted flow cytometry analysis on CaSki cells subjected to ENB101-LNP, cisplatin, and a combination treatment of both (Fig 1E and 1F). The analysis revealed that independent treatments with ENB101-LNP and cisplatin each induced a surge in the expression of HLA class I within CaSki cells. When both treatments were administered simultaneously, a synergistic effect was observed, leading to a further increase in HLA class I expression. These findings highlight the significant impact of ENB101-LNP, whet her used alone or in combination with cisplatin, on the expression of essential proteins and its synergistic effect with cisplatin in modulating HLA class I expression. Regarding PD-L1 expression, cisplatin treatment led to an increase, while ENB101-LNP treatment did not significantly affect PD-L1 expression compared to the control. When both treatments were combined, PD-L1 levels resembled those treated with cisplatin alone, indicating that the inhibition of E7 by ENB101-LNP had a minimal impact on decreasing PD-L1 expression.

### ENB101-LNP and cisplatin synergistically inhibit tumor growth in a CaSki xenograft mouse model

**Fig 2:**
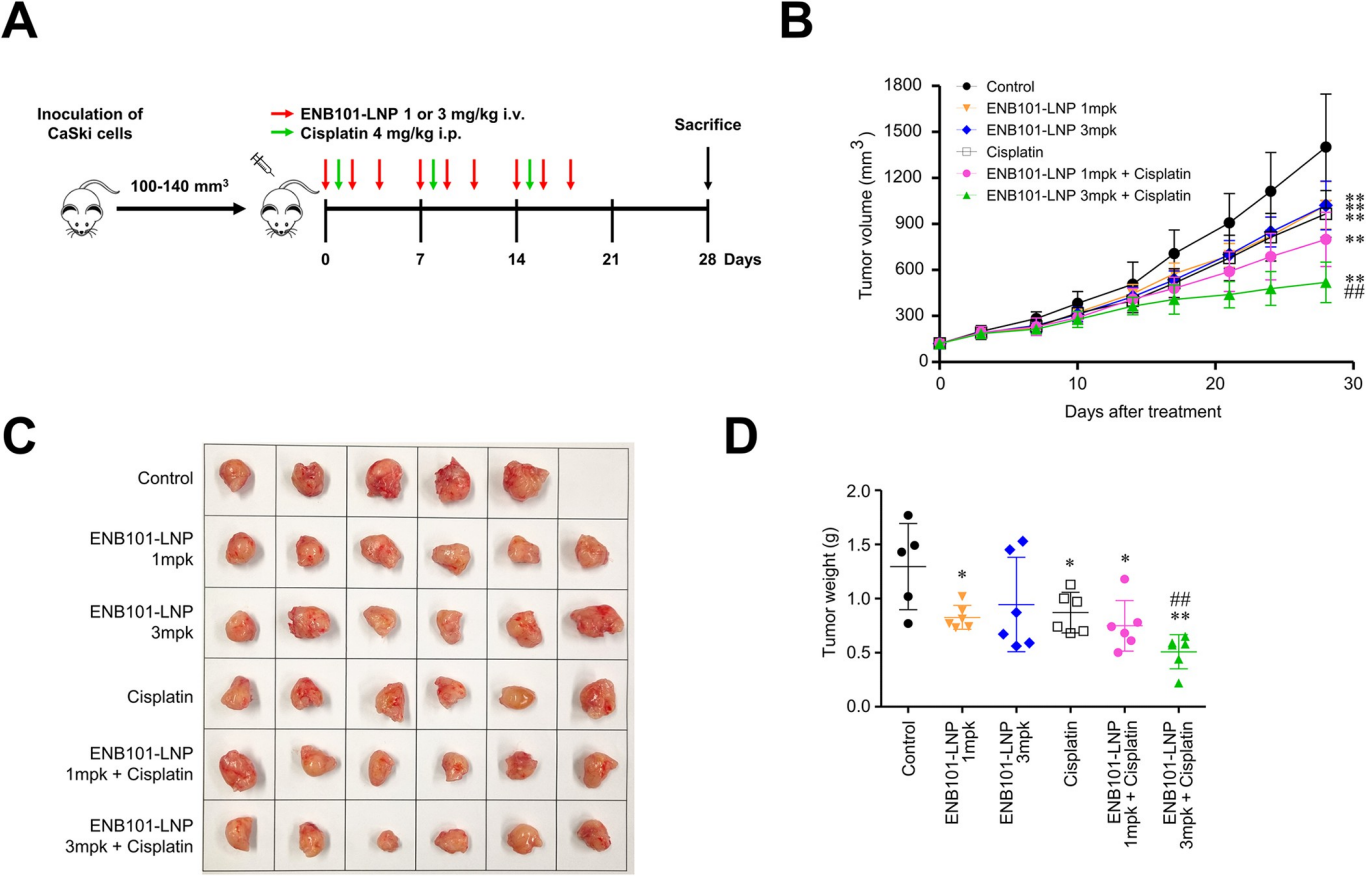
ENB101-LNP and cisplatin synergistically inhibit tumor growth in a CaSki xenograft mouse model. CaSki cells were injected into six-week-old female BALB/c nude mice (5 × 10^6^ cells/mouse). Day 0 corresponds to 2 weeks after inoculation of CaSki cells when the tumor volume reached 100–140 mm^3^. The mice were randomly divided into six groups (n = 6 per group): (1) blank control; (2) 1 mg/kg ENB101-LNP; (3) 3 mg/kg ENB101-LNP; (4) cisplatin; (5) 1 mg/kg ENB101-LNP-cisplatin; (6) 3 mg/kg ENB101-LNP-cisplatin. ENB101-LNP was administered via intravenous injection three times weekly for a duration of 3 weeks, while cisplatin was administered via intraperitoneal injection once a week for 3 weeks. (B) Tumor growth curves were plotted to calculate the tumor inhibition rate. The average tumor volumes were calculated and presented as mean ± standard deviation (Wilcoxon test, ** P < 0.01; compared with control, ## P < 0.01; compared with cisplatin). Tumors were dissected from CaSki cell xenograft mice (C), weighed, and the results were analyzed using the unpaired *t*-test (* P < 0.05, ** P < 0.01, ## P < 0.01) (D).

We evaluated the efficacy of ENB101-LNP and cisplatin using a xenograft model of cervical cancer by subcutaneously injecting CaSki cells into the right thigh of BALB/c nude mice. The mice were divided into different groups and received the drugs according to specific dosages and administration schedules outlined in Fig 2A. ENB101-LNP was administered into the tail vein three times a week, with a two-day interval between each administration, for a total of nine administrations. The cisplatin was administered intraperitoneally once a week for a total of three doses. Tumor volume was measured twice a week for all mice, including during the group separation period. The average and standard deviation of tumor volumes measured after Day 0 are presented in Fig 2B. At the end of the 28-day experiment, the control group displayed a tumor volume of 1,401 ± 346 mm^3^. The ENB101-LNP administration groups at 1 mg/kg and 3 mg/kg showed tumor volumes of 1,021 ± 154 mm^3^ and 1,022 ± 158 mm^3^, respectively. The cisplatin administration group (4 mg/kg) had a tumor volume of 966 ± 152 mm^3^. The combination administration groups of ENB101-LNP at 1 mg/kg with cisplatin at 4 mg/kg and ENB101-LNP at 3 mg/kg with cisplatin at 4 mg/kg exhibited tumor volumes of 800 ± 176 mm^3^ and 520 ± 131 mm^3^, respectively.

After the mice were euthanized, the tumor tissues were removed, weighed, and photographed. S2A Fig shows the photographs of each group of mice with implanted tumors, and Fig 2C shows the photographs of harvested tumors. The tumor growth inhibition (TGI) value was calculated using the formula TGI = (1—T/C) × 100, where T represents the mean tumor volume of the treated group and C represents the mean tumor volume of the control group. Compared to the control and other treatment groups, a significant inhibition of tumor growth was observed in the ENB101-LNP with cisplatin group. The tumor inhibition rates were 29.7% for 1 mg/kg ENB101-LNP (P = 0.032), 29.6% for 3 mg/kg ENB101-LNP (P = 0.032), 34.0% for cisplatin (P = 0.01), and 47.0% for 1 mg/kg ENB101-LNP with cisplatin (P < 0.001). Notably, the combination of 3 mg/kg ENB101-LNP with cisplatin exhibited superior antitumor efficacy, with a tumor inhibition rate of 68.8% (P < 0.001). TGI of 3 mg/kg ENB101-LNP with cisplatin showed a statistically significant difference from the control group and also exhibited a statistically significant difference compared to the cisplatin-only group. Consistently, the synergistic antitumor effect of ENB101-LNP and cisplatin was confirmed through the results of the tumor weights (Fig 2D).

### Combining ENB101-LNP with cisplatin enhances apoptotic effects in xenograft tumors

**Fig 3:**
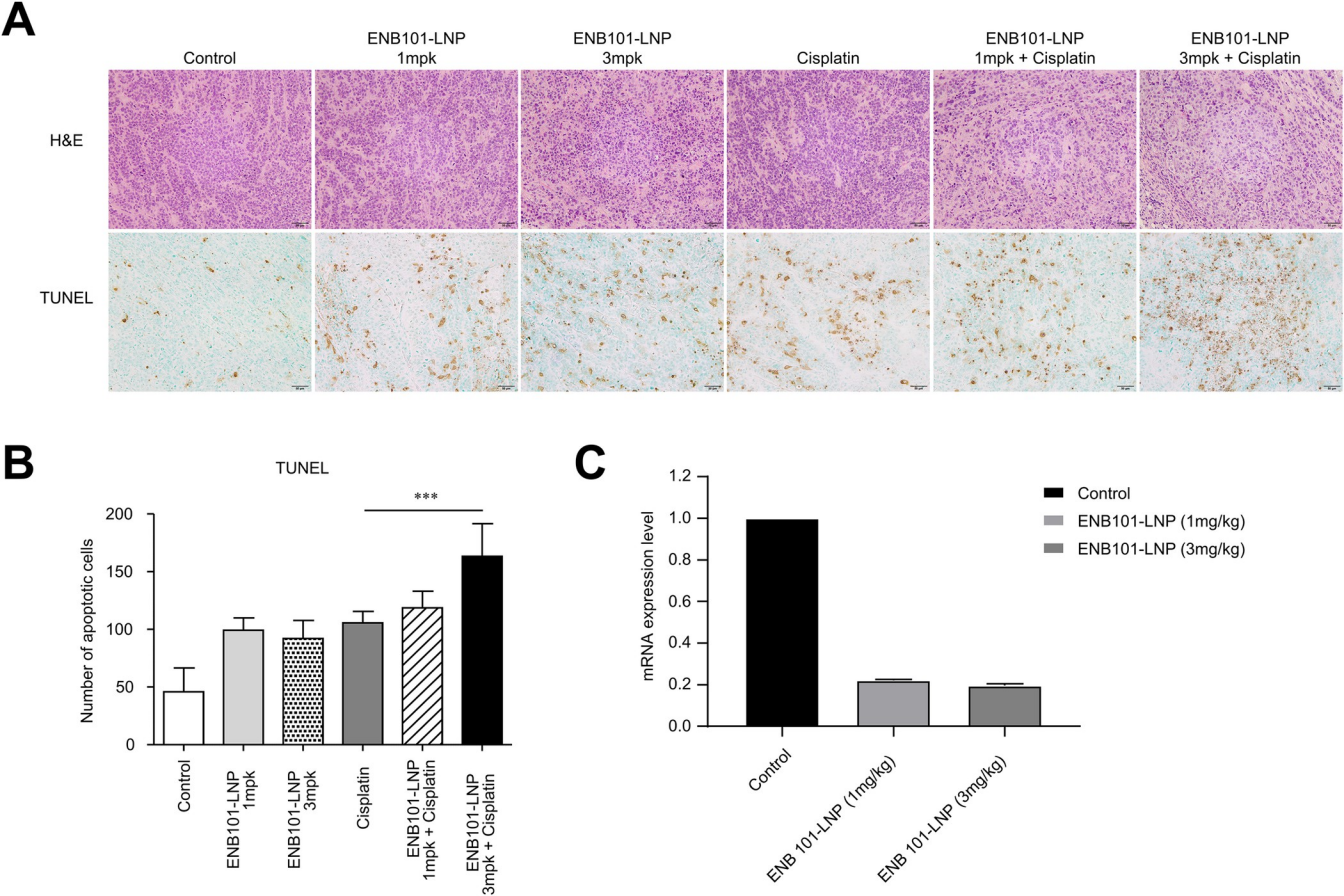
Combining ENB101-LNP with cisplatin enhances apoptotic effects in xenograft tumors. (A) Histological H&E staining of xenograft tumors (top panels, original magnification × 200) and TUNEL staining of apoptotic xenograft tumor cells in the six groups (bottom panels, original magnification × 200). (B) Quantitative result of TUNEL assay (unpaired *t*-test, *** P < 0.001). (C) Tumor tissues were collected from CaSki xenograft mice after 6 injections on day 11. The expression levels of HPV16 E7 were detected in tumor tissues using qRT-PCR.

Tumors treated with ENB101-LNP and cisplatin exhibited significant increases in TUNEL-positive cells (Fig 3A and 3B). Successful knockdown of HPV16 E6/E7 mRNA expression levels was confirmed in both the 1 mg/kg and 3 mg/kg ENB101-LNP groups, suggesting that the observed increase in apoptotic cells may be attributed to the restoration of TP53 and RB (Fig 3C).

### ENB101-LNP combined with irradiation inhibits tumor growth in xenograft tumors

**Fig 4:**
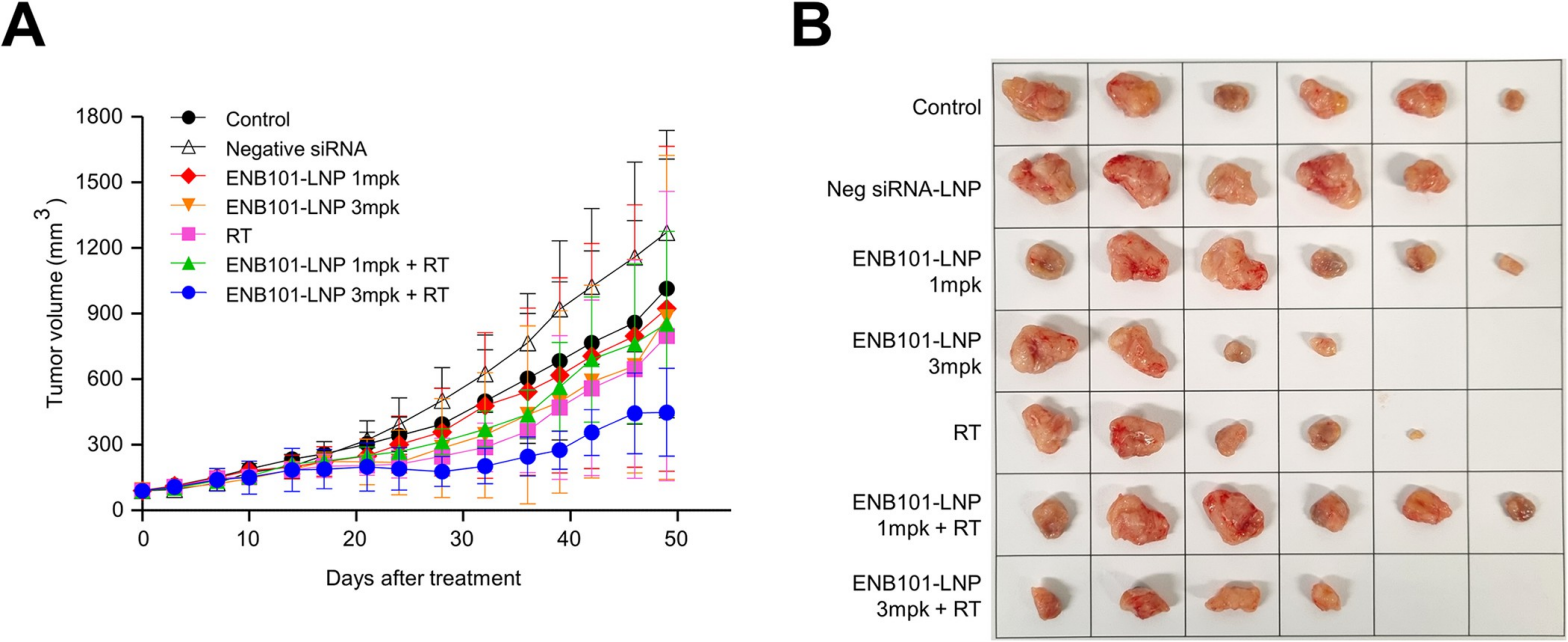
ENB101-LNP combined with irradiation inhibits tumor growth in xenograft tumors. Nude mice bearing CaSki xenografts were randomly divided into seven groups (n = 6 per group): (1) blank control; (2) Negative siRNA-LNP; (3) 1 mg/kg ENB101-LNP; (4) 3 mg/kg ENB101-LNP; (5) RT; (6) 1 mg/kg ENB101-LNP-RT; (7) 3 mg/kg ENB101-LNP-RT. ENB101-LNP was administered via intravenous injection three times weekly for 3 weeks, while RT was administered at a single dose of 5 Gy irradiation on the day after the first administration of ENB101-LNP. (A) Tumor growth curves were plotted, and the tumor growth inhibition was calculated for each group. (B) Representative images of CaSki tumors from nude mice at the experimental endpoint (49 days after treatment).

We then evaluated whether the combination of ENB101-LNP and radiation therapy, which is one of the mainstay treatments for cervical cancer along with cisplatin, could lead to enhanced therapeutic effects against cervical cancer. BALB/c nude mice were subcutaneously injected CaSki cells and were randomly divided into seven groups for ENB101-LNP treatment. ENB101-LNP was administered intravenously three times per week for three weeks, and a single dose of 5 Gy radiation was given on the day following the first ENB101-LNP injection. The mice were euthanized 49 days post-treatment initiation, at which point their tumor tissues were subjected to analysis (S3A and S3B Fig). The tumors, dissected from the xenograft mice, are depicted in Fig 4B. Throughout the treatment period, no significant differences were observed in the mice’s weight relative to the method of treatment employed (S3C Fig). The tumor volume, according to the treatment schedule, is presented in Fig 4A. The results showed that the TGI rate was 10.0% in the 1 mg/kg ENB101-LNP group, 14.4% in the 3 mg/kg ENB101-LNP group, 23.5% in the radiation group, 17.4% in the 1 mg/kg ENB101-LNP-RT group, and 61.2% in the 3 mg/kg ENB101-LNP-RT group. Notably, the combination of 3 mg/kg ENB101-LNP with radiation therapy exhibited superior antitumor efficacy, with a tumor inhibition rate of 61.2%. These findings highlight the potential of combining ENB101-LNP with radiation therapy to achieve improved outcomes in the treatment of cervical cancer.

### Synergistic effect of ENB101-LNP and cisplatin on the expression of p53, p21, RB, HLA-A, and PD-L1 in xenograft tumors

**Fig 5:**
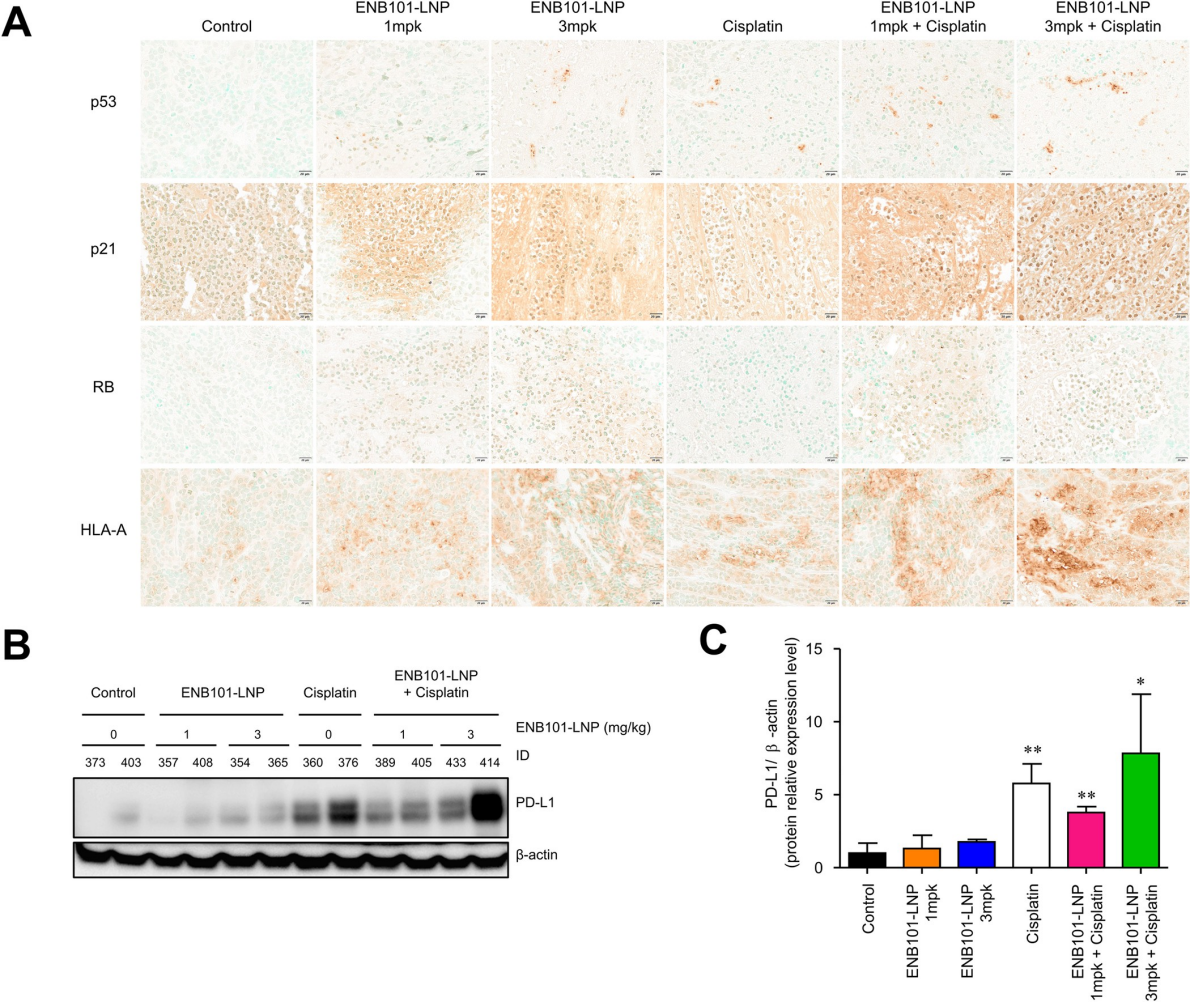
Synergistic effect of ENB101-LNP and cisplatin on the expression of p53, p21, RB, HLA-A, and PD-L1 in xenograft tumors. (A) Representative IHC images showing the expression levels of p53 (top panel), p21 (second panel), RB (third panel), and HLA-A (bottom panel) on tumor tissues of each group (original magnification × 200). (B, C) Western blotting analysis of PD-L1 and β-actin protein levels in CaSki xenografts. The density of the band was measured using Image J software (unpaired *t*-test, * P < 0.05, ** P < 0.01).

To examine changes in protein expression levels, we performed immunohistochemistry on tumor tissues obtained after each treatment (Fig 5A). The ENB101-LNP treatment group showed increased expression of p53 and p21 compared to the control and cisplatin alone groups. In terms of RB expression, the ENB101-LNP treatment group showed an increase, while the cisplatin treatment group exhibited a pattern similar to that of the control group. Additionally, we analyzed the ability of ENB101-LNP to upregulate the expression of HLA-A molecules on CaSki cells. HLA-A expression was more intense in the ENB101-LNP and cisplatin treatment groups compared to the control group, with the strongest degree of staining observed in the group that received ENB101-LNP 3 mg/kg combined with cisplatin. Our findings provide evidence that treatment with ENB101-LNP, either alone or in combination with cisplatin, enhances the antigen-specific recognition of CaSki cells by cytotoxic T lymphocytes. This effect can be attributed, at least in part, to improved antigen presentation on the cell surface resulting from increased transcription of HLA class I molecules in the treated cells. Western blot analysis revealed that while the cisplatin group showed increased expression of PD-L1, the combination treatment of ENB101-LNP and cisplatin displayed decreased expression of PD-L1 compared to cisplatin alone, although the difference was not statistically significant (Fig 5B and 5C).

## Discussion

Targeted therapies against HPV E6/E7 oncogenes have shown promising results in eliminating malignant cells in cervical carcinoma. In addition, novel genetic approaches and genome-editing systems offer potential strategies for treating cervical cancer by specifically targeting and removing HPV E6/E7 genes [21, 29]. Among these approaches, RNAi has emerged as a promising therapy that can specifically target HPV E6/E7 and induce apoptosis or senescence in cancer cells [13, 16, 17]. Previous studies have demonstrated that combining siRNA targeting E6/E7 with cisplatin or radiation therapy can restore TP53 and RB/E2F, resulting in improved therapeutic outcomes for cervical cancer *in vivo* [14, 19]. However, delivering synthesized RNA molecules to the cytoplasm can present challenges, resulting in varying levels of the target RNA molecule and heterogeneous outcomes [30]. The stability and resistance to degradation of RNA structures can also vary, which can affect their therapeutic effectiveness [31]. Additionally, exogenous RNA molecules may contain motifs that can activate immune responses, potentially resulting in severe adverse reactions [32].

To overcome these challenges, researchers have focused on developing efficient delivery strategies to protect RNA molecules and facilitate their targeted transportation to specific tissues. Non-viral nanoparticles, particularly LNPs made from polymers and lipids, have gained attention in this field [31–34]. LNPs possess favorable physical and chemical properties, including size, shape, surface characteristics, and interactions with anionic molecules, making them highly suitable as delivery carriers for active pharmaceutical ingredients in medicine [35]. LNPs offer advantages such as protecting RNA from degradation, facilitating escape from acidic endosomes, and providing a high payload capacity compared to polymers [36]. Consequently, LNPs have played a crucial role in the advancement of cancer nanomedicines, allowing RNA molecules to become a potent therapeutic tool for treating cancer [37].

In this study, we aimed to evaluate the anti-cancer effects of ENB101-LNPs, which are ionizable lipid nanoparticles containing siRNA that targets HPV 16 E6/E7 oncogenes. Through comprehensive *in vitro* and *in vivo* investigations, we have unequivocally demonstrated the remarkable anti-cancer activity of ENB101-LNP against HPV16 E6/E7. Specifically, treatment with ENB101-LNP effectively suppressed the expression of HPV16 E6/E7 in CaSki cells, resulting in the restoration of p53 and p21 protein levels. This inhibition resulted in the induction of apoptosis. Previous studies have revealed the p53-dependent cell cycle arrest and apoptosis [38, 39]. Furthermore, our *in vivo* experiments employing a CaSki xenograft mouse model provided compelling evidence of the therapeutic efficacy of ENB101-LNP. Treatment with ENB101-LNP as monotherapy significantly inhibited tumor growth, reduced HPV16 E6/E7 expression, and restored the levels of tumor-suppressing proteins. Importantly, when ENB101-LNP was combined with cisplatin, a synergistic effect was observed, leading to further enhanced therapeutic outcomes. These findings underscore the potential synergy of combining ENB101-LNP with cisplatin, highlighting the significant therapeutic value achieved through this combination.

Cervical cancer is often characterized by disrupted expression of HLA class I molecules, which can affect the presentation of HPV-derived antigenic peptides to cytotoxic T cells [40, 41]. In our study, we observed a synergistic effect on HLA-A expression when combining ENB101-LNP and cisplatin, as demonstrated in both *in vitro* and *in vivo* analyses. The observed increase in HLA-A expression indicates that CaSki cells have an enhanced ability to present antigens, which could potentially result in improved recognition by cytotoxic T lymphocytes. Notably, the group treated with ENB101-LNP 3 mpk in combination with cisplatin exhibited the most pronounced effect. Overall, our results suggest that the combined treatment of ENB101-LNP and cisplatin not only inhibits cell proliferation but also enhances HLA-A expression, promoting antigen presentation and stimulating an immune response against cervical cancer cells. These findings underscore the potential of ENB101-LNP as an immunotherapeutic agent for cervical cancer.

Regarding PD-L1 expression, our study yielded contrasting results compared to previous research. Previous studies have indicated that cisplatin-based chemotherapy can increase PD-L1 expression in various types of cancer, including cervical cancer [42–44]. The upregulation of PD-L1, along with the presence of an immune-rich microenvironment after chemotherapy, suggests the potential application of PD-1/PD-L1 inhibitors in the neoadjuvant setting. Interestingly, a significant positive correlation has been reported between HPV16-E7 and PD-L1 protein expression in cervical cancer tissues [45]. Therefore, we expected that the downregulation of E6/E7 by ENB101-LNP would lead to a reduction in PD-L1 expression. In both our *in vitro* and *in vivo* experiments, we observed that treatment with cisplatin resulted in the anticipated upregulation of PD-L1 expression, consistent with our initial expectations. However, the combination treatment of ENB101-LNP did not significantly affect the levels of PD-L1 compared to the control group. This unexpected finding suggests that the inhibitory effect of ENB101-LNP on E6/E7 had a limited impact on reducing PD-L1 expression. Further investigations are required to fully comprehend the underlying mechanisms and implications of these observations. Moreover, these findings have important implications for immunotherapy in cervical cancer.

Our study has several limitations that should be acknowledged. First, it is important to note that the direct applicability of our findings from a mouse model to human patients may be limited, and further investigations are needed to assess the clinical feasibility and effectiveness of using ENB101-LNP with cisplatin as a treatment for cervical cancer in humans. Second, although we evaluated alterations in protein expression levels, additional mechanistic studies are necessary to fully elucidate the underlying molecular pathways and mechanisms underlying the observed effects. Additionally, it is crucial to conduct a comprehensive evaluation of the safety profile, potential side effects, and long-term consequences associated with the administration of ENB101-LNP and cisplatin as a treatment for cervical cancer. Despite these limitations, our study provides a strong basis for future investigations that aim to evaluate the practicality and potential clinical applications of siRNA-LNP strategies in treating cervical cancer. With continued research and development, siRNA-LNP therapy, including ENB101-LNP, has significant potential as an effective and targeted treatment option for cervical cancer.

In conclusion, our study provides valuable insights into the potential therapeutic application of ENB101-LNPs, which are ionizable lipid nanoparticles containing siRNA that targets HPV 16 E6/E7 oncogenes, for the treatment of cervical cancer. Importantly, our findings emphasize the observed synergistic effect when combining ENB101-LNPs with cisplatin. This combination approach demonstrates the ability to selectively silence viral oncogenes and restore the function of tumor suppressor genes, as evidenced in our mouse model of cervical cancer.

## Supporting information

S1 Fig Relative protein expression in CaSki cells treated with ENB101-LNP and cisplatin. Relative levels of (A) HPV16 E7, (B) p53 and p21 proteins were determined after adjusting for β-actin.
(TIF)

S2 Fig Subcutaneous CaSki xenografts treated with ENB101-LNP and cisplatin in nude mice. (A) Representative images of mice on day 28 after ENB101-LNP and cisplatin treatment. (B) Trends in body weights of the mice during the periods of ENB101-LNP and cisplatin administration.
(TIF)

S3 Fig Subcutaneous CaSki xenografts treated with ENB101-LNP and RT in nude mice. (A) Typical images of mice 49 days after ENB101-LNP and RT treatment. (B) The tumor tissues were resected from the mice at the experimental endpoint. The weights of the tumors in each group were measured. (C) There were no significant changes in the body weights of the mice during the periods of ENB101-LNP and RT treatment.
(TIF)

S1 File Raw data for graphs. (XLSX)

S2 File Cell_line STR analysis report. (PDF)

S1 Raw images (TIF)
