# Supplementary figures and images for: Evaluation of the anti-cancer efficacy of lipid nanoparticles containing siRNA against HPV16 E6/E7 combined with cisplatin in a xenograft model of cervical cancer
Source: PLoS One. 2024 Feb 16;19(2):e0298815. doi: 10.1371/journal.pone.0298815 (PMC10871510; doi:10.1371/journal.pone.0298815)

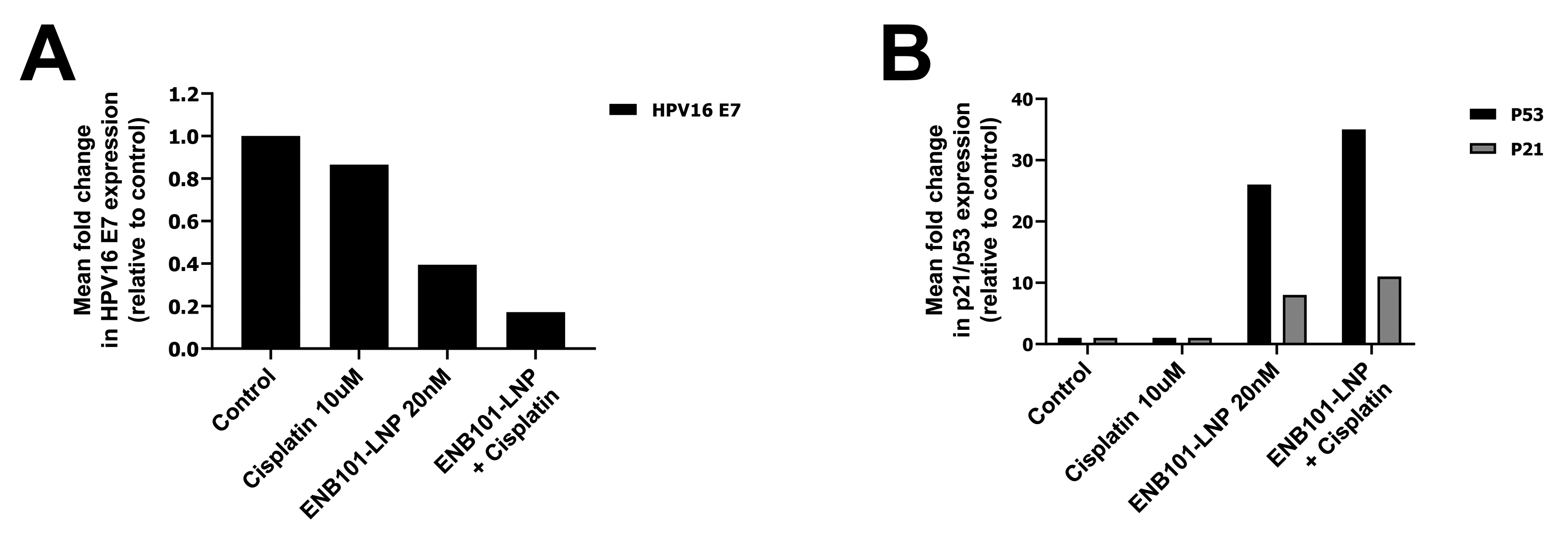

Supplement: S1 Fig — Relative levels of (A) HPV16 E7, (B) p53 and p21 proteins were determined after adjusting for β-actin. (TIF) [file pone.0298815.s001.tif]

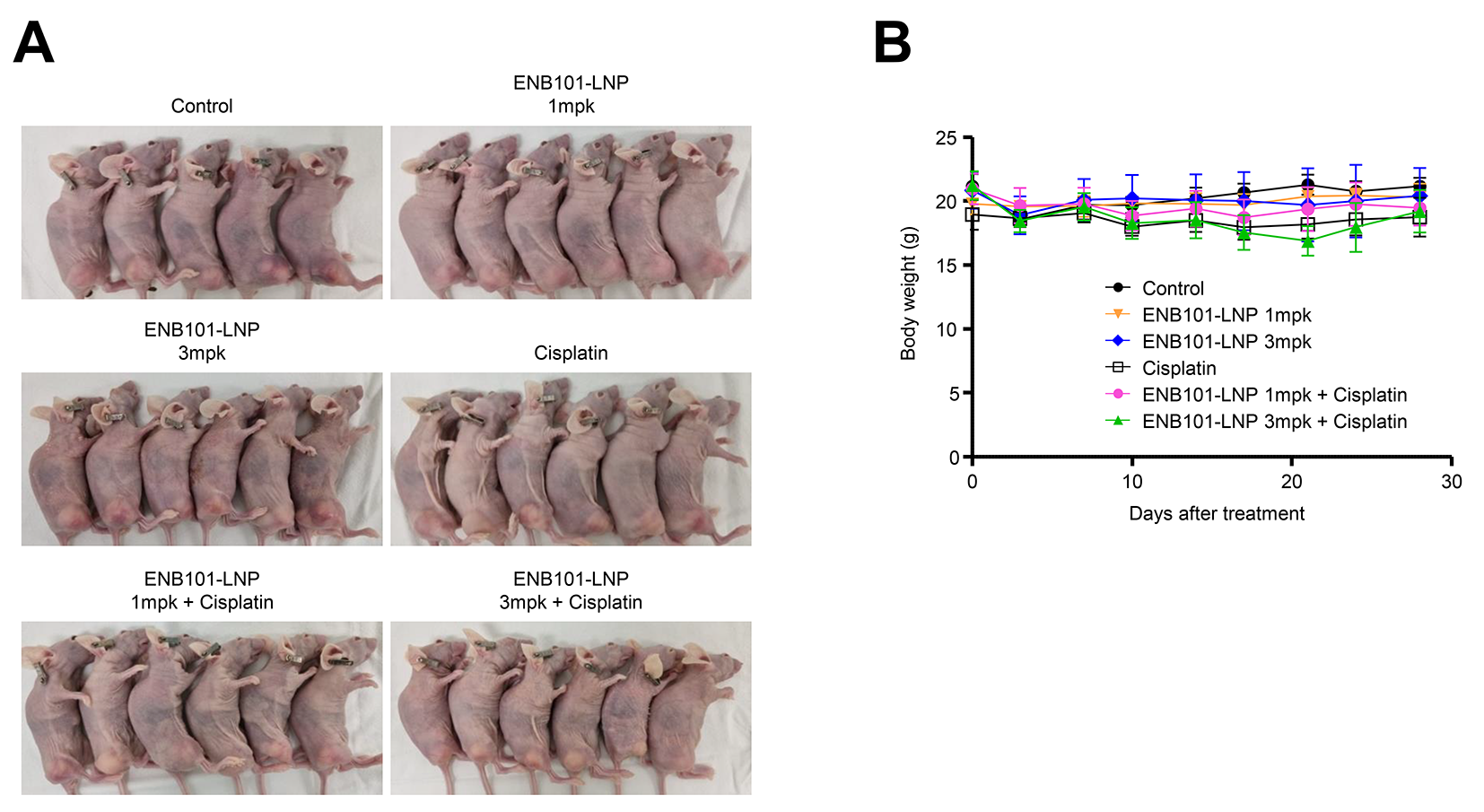

Supplement: S2 Fig — (A) Representative images of mice on day 28 after ENB101-LNP and cisplatin treatment. (B) Trends in body weights of the mice during the periods of ENB101-LNP and cisplatin administration. (TIF) [file pone.0298815.s002.tif]

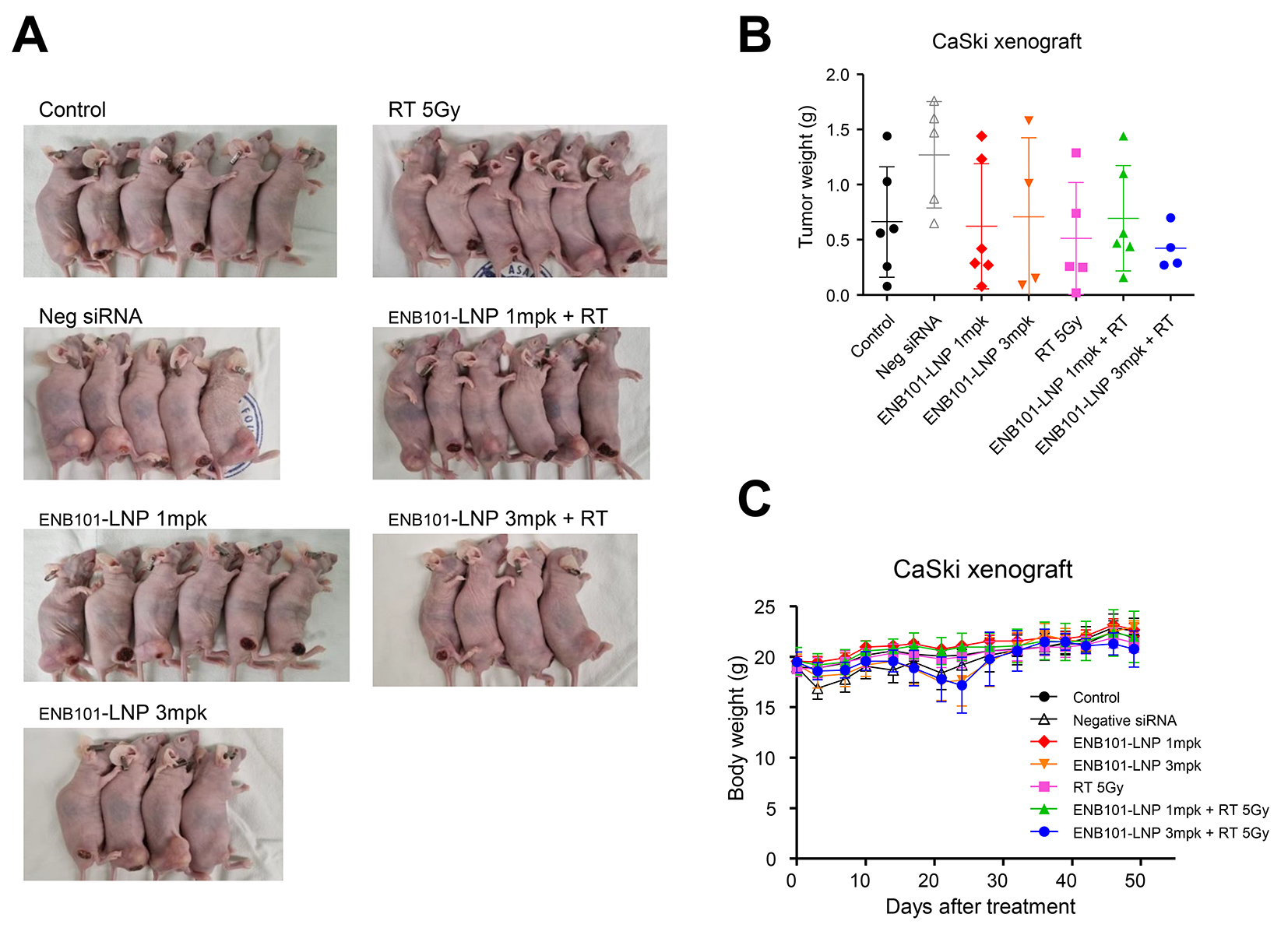

Supplement: S3 Fig — (A) Typical images of mice 49 days after ENB101-LNP and RT treatment. (B) The tumor tissues were resected from the mice at the experimental endpoint. The weights of the tumors in each group were measured. (C) There were no significant changes in the body weights of the mice during the periods of ENB101-LNP and RT treatment. (TIF) [file pone.0298815.s003.tif]

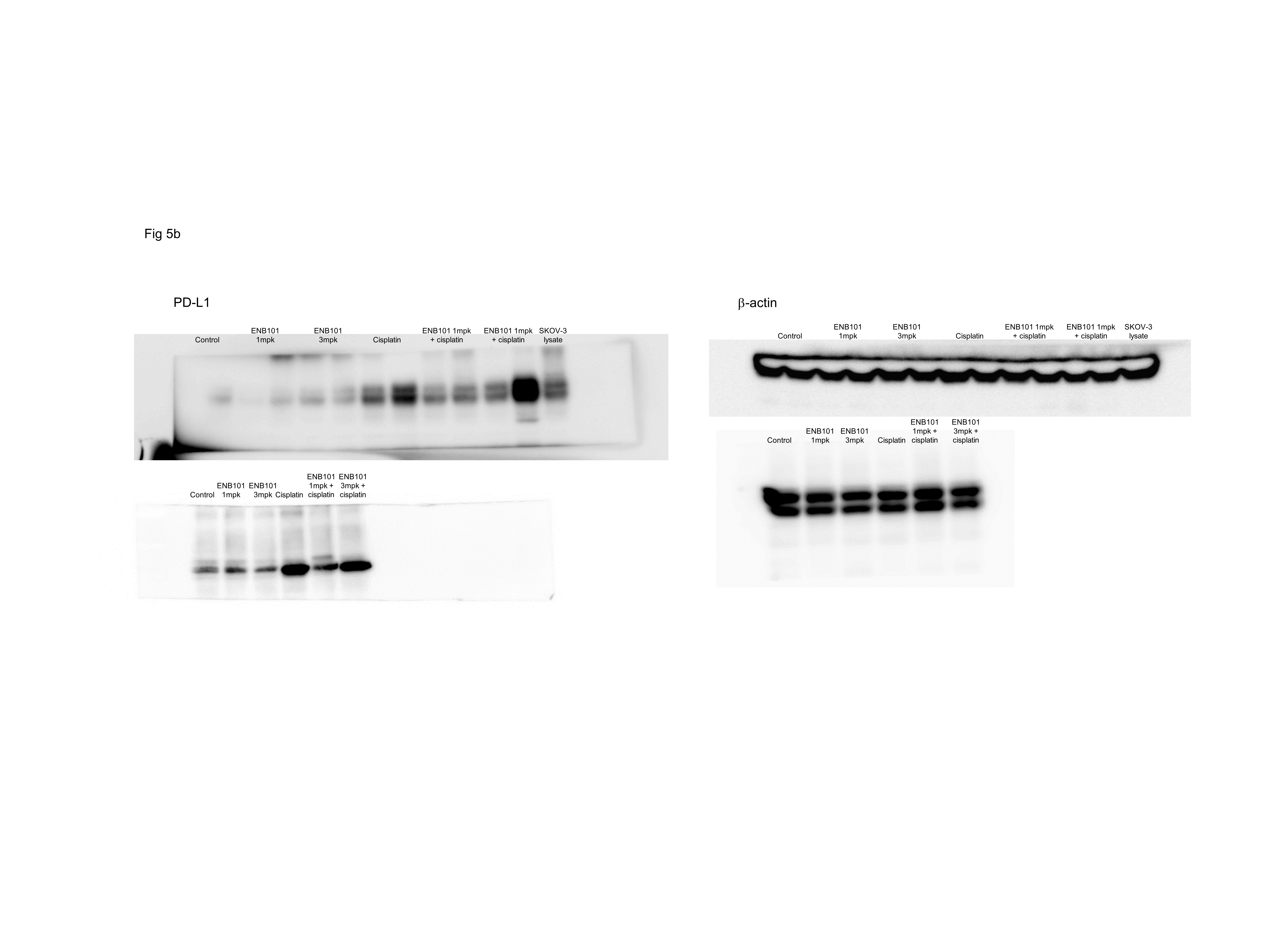

Supplement: S1 Raw images — (TIF) [file pone.0298815.s006.tif]
