# Supplementary material for: Evaluation of the anti-cancer efficacy of lipid nanoparticles containing siRNA against HPV16 E6/E7 combined with cisplatin in a xenograft model of cervical cancer
Source: PLoS One. 2024 Feb 16;19(2):e0298815. doi: 10.1371/journal.pone.0298815 (PMC10871510; doi:10.1371/journal.pone.0298815)

Humanizing Genomics macrogen

CES CAPILLARY ELECTROPHORESIS SEQUENCING

Cell line STR

Analysis Result Report

INFORMATION

Order No.	HC00365332
Date	2021. 10. 28
Sample Type	Cell line

METHOD

- The AmpFlSTR® Identifiler® PCR Amplification Kit is a short tandem repeat (STR) multiplex assay that amplifies 15 tetranucleotide repeat loci and the Amelogenin gender-determining marker in a single PCR amplification:

  - All thirteen of the required loci for the Combined DNA Index System (CODIS) (Budowle et al., 1998).
  - Two additional loci, D2S1338 and D19S433.

- We use Applied Biosystems® 3730/3730xl DNA Analyzer and analyze data using GeneMapper ID v3.2. For reliability, Positive (DNA9947A) and Negative are used together during the test.

- We do experiment based on the Genotype Identification Kit of ABI which is international standard method, and we also obtained a method using PowerPlex21 of Promega to secure diversity.

REFERENCE

- Budowle, B. et al. 1998. CODIS and PCR Based Short Tandem Repeat Loci: Law Enforcement Tools. Second European Symposium on Human Identification. 73–88.
- Holt, C., Stauffer, C., Wallin, J., Lazaruk, L., Nguyen, T., Budowle, B., and Walsh, P. 2000. Practical applications of genotypic Surveys for forensic STR testing. Forensic Sci.
- Butler, J.M. 2005. Forensic DNA Typing. Burlington, MA:Elsevier Academic Press.

PROCESS

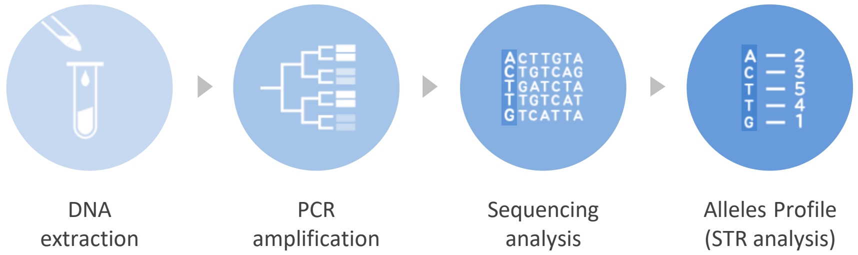

NOTE

- **Cell line authentication?**

  Cell line authentication Service is genetic analyzing service using Multiplex-PCR Kit which is comprised of STR(Short Tandem Repeat) loci.

- **STR Marker Information (AmpFlSTR®Identifiler® PCR Amplification KIT)**

STR Locus	Label	Size Range	Chromosomal Location	Repeat Sequence 5'→3'
D8S1179	6-FAM	123-170	8q24.13	(TCTA)(TCTG)
D21S11		185-239	21q21.1	(TCTA)(TCTG)
D7S820		255-291	7q21.11	GATA
CSF1PO		305-342	5q33.1	TAGA
D3S1358	VIC	112-140	3p21.31	(TCTA)(TCTA)
TH01		163-202	11p15.5	TCAT
D13S317		217-245	13q31.1	TATC
D16S539		252-292	16q24.1	GATC
D2S1338		307-359	2q35	(TGCC)(TTCC)
Amelogenin	PET	107,113	XP22.22/YP11.2	NA
D5S818		134-172	5q23.2	AGAT
FGA		215-355	4q31.3	CTTT
D19S433	NED	102-135	19q12	AAGG
vWA		155-207	12q13.31	(TCTG)(TCTA)
TPOX		222-250	2p25.3	GAAT
D18S51		232-345	18q21.33	AGAA

Cell line authentication

Result Report

Do date.	2021. 10. 28	Method	A-STR Genotyping analysis, 3730XL DNA analyzer
Reagent	AmpFISTR®Identifiler® KIT	Percent Match	100 %

Cell name	CaSki (Reference)		APEX_CaSki	
Amelogenin	X	X	X	X
STR Locus	Allele1	Allele2	Allele1	Allele2
D8S1179			15	15
D21S11			30	30
D7S820	8	11	8	11
CSF1PO	10	10	10	10
D3S1358	15	15	15	15
TH01	7	7	7	7
D13S317	8	12	8	12
D16S539			11	12
D2S1338			21	21
D5S818	13	13	13	13
FGA	21	21	21	21
D19S433			15	16
vWA	17	17	17	17
TPOX	8	8	8	8
D18S51			17	17

Interpretation of Results

** Reference : ICLAC - Match Criteria for Human Cell Line Authentication*

1 Is the percent match result in the range 0 - 55% ?

- This result is consistent with the two samples being unrelated (different donors)

2 Is the percent match result in the range 56 - 79% ?

- This result is indeterminant and may need further testing

3 Is the percent match result in the range 80 - 100% ?

- This result is consistent with the two samples being related (same donor)

*This report is for research purposes only

Raw Data Guide

① Sample name
② STR amplification kit
③ STR marker
④ X axis: Fragment size
⑤ Y axis: Relative fluorescence units (RFU)
⑥ STR repeat number (13 = 13 repeats)
⑦ Decimal point (9.3 = ATGC ATGC …. ATGC ATG)
⑧ sex determining marker (Female: XX / Male: XY)

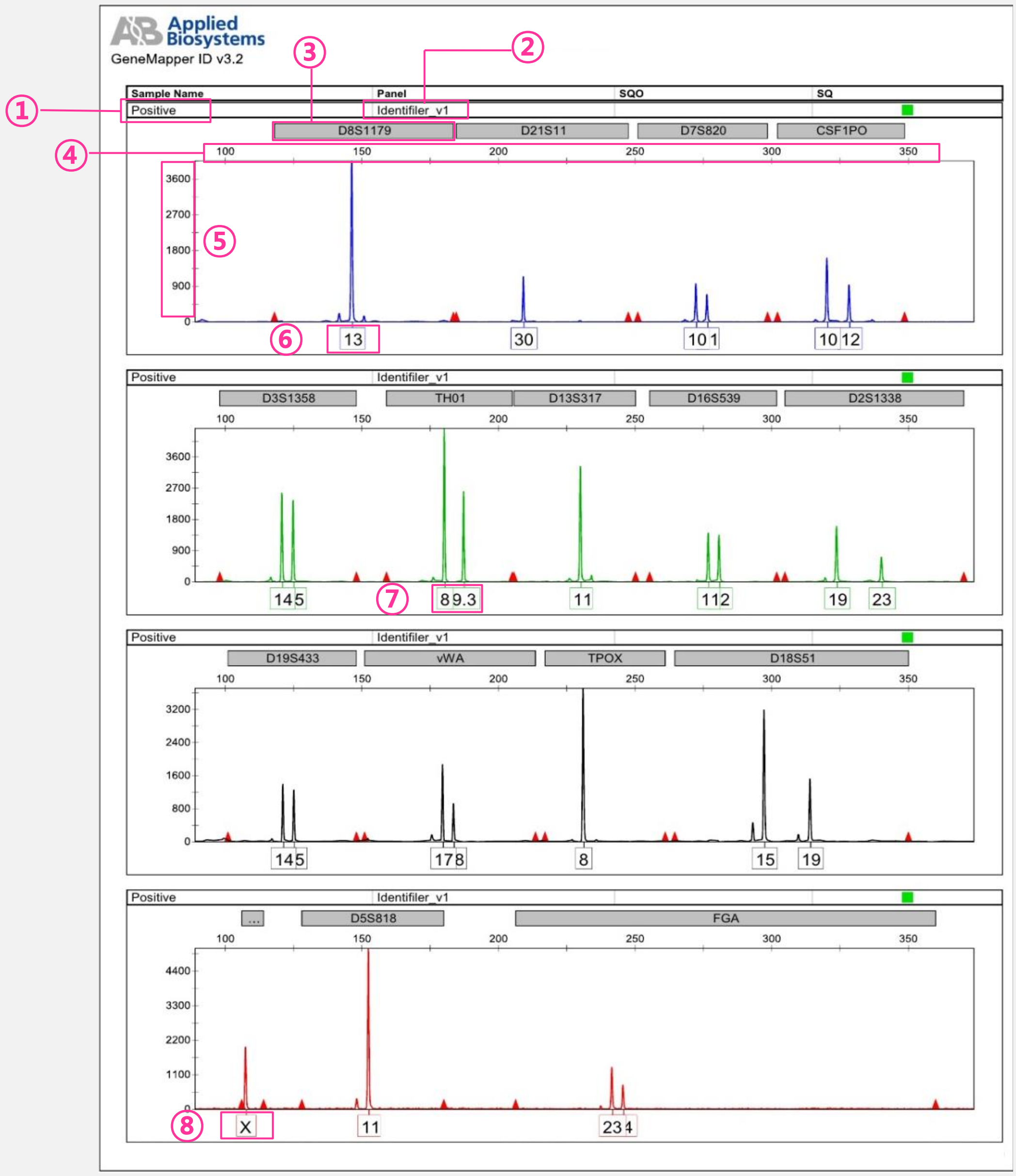

Supplement: S2 File — (PDF) [file pone.0298815.s005.pdf]
